# A comprehensive investigation of Bi_2_O_3_ on the physical, structural, optical, and electrical properties of K_2_O.ZnO.V_2_O_5_.B_2_O_3_ glasses

**DOI:** 10.1038/s41598-024-58567-w

**Published:** 2024-04-12

**Authors:** S. Ibrahim, A. A. Ali, Ahlam M. Fathi

**Affiliations:** 1 Glass Research Department, National Research Centre, El-Buhouth St., Dokki, Giza, 12622 Egypt; 2 Physical Chemistry Department, National Research Centre, El-Buhouth St., Dokki, Giza, 12622 Egypt

**Keywords:** Bi_2_O_3_, Borovanadate glasses, FTIR, Optical properties, Urbach energy, Electrical properties, Materials science, Optics and photonics

## Abstract

The multi-component glass system has a composition of 10K_2_O–10ZnO–55 B_2_O_3_–(25–x)V_2_O_5_–xBi_2_O_3_ (x = 4, 5, 7.5, 9, 10 mol%) are synthesized by the melt-quenching method. Using X-ray diffraction examination, the amorphous phase in the material was confirmed. The physical characteristics of the produced compositions are examined using density (D) and molar volume (V_m_). Calculations of physical properties showed that adding Bi_2_O_3_ from 4 to 10 mol% increased the glass density from 2.7878 to 3.3617 g cm^−3^ and decreased the molar volume from 40.4196 to 38.5895 cm^3^/mol. Studies of glass samples using the FTIR show bands of absorption for oxides in different structural groups. Octahedral [${{\text{BiO}}}_{6}$], [${{\text{BO}}}_{4}$], and tetrahedral [${{\text{BO}}}_{3}$] structural units are observed in the present glass matrices. The cutoff wavelength (${\lambda }_{C}$), and optical band gap energy were determined using UV absorption spectra. The increase in non-bridging oxygens can be linked to the decrease in optical band gap energy (${E}_{opt}$) (direct and indirect) and the increase in cutoff wavelength with an increase in Bi_2_O_3_ content. This is attributed to the existence of bismuth ions and the creation of non-bridging oxygens. Besides that, the values of optical parameters, viz., optical electronegativity, refractive index, and molar refractivity, are calculated. The metallization criterion values are less than 1 and the glass samples exhibit an increased tendency towards metallization. Both the conductivity and the dielectric constant increase with the rise in Bi_2_O_3_ content, however, the dielectric loss and the impedance reduce. The behavior and values of conductivity for the studied glasses reveal the semiconducting properties of all glass samples. These results suggest that the produced glass samples may be employed as amorphous semiconductors in electronics and memory switching devices.

## Introduction

Glass, an isotropic material, offers many benefits over crystalline materials, including being inexpensive, easy to fabricate, and having no grain boundaries^1^. The oxide glass family is quite large and is always evolving. Oxide glasses are used in several well-known and highly technical fields, including X-ray protection, fibre optic equipment, and laboratory glassware. Network formers such as silicate, vanadate, borate, borovanadate, and borosilicate, as well as network modifiers like transition metals, alkali, and alkaline earth, make up the oxide glasses^2^.

Borate glasses are the most effective in forming glass out of all the varieties of glasses. Because of its greater binding strength, smaller cationic size, and lower heat of fusion, B_2_O_3_ is a useful glass-forming material^3^. Glass formation at low temperatures is easy because of its excellent thermal stability and chemical durability^4^. It demonstrates excellent mechanical stability, optimal bandwidths, better infrared transmissions, and great photonic characteristics^5^. In borate glasses, the B^3+^ atom often coordinates with 3 or 4 oxygen atoms to create [${{\text{BO}}}_{3}$] or [${{\text{BO}}}_{4}$] structural units^6^.

Vanadium oxide, which has good mechanical and optical characteristics, is one of the newest compounds incorporated into the B_2_O_3_-based glass. Because they occur in glass networks simultaneously in various coordination’s (i.e., ${VO}_{4 } \;\; and \;\; {VO}_{5}$) as well as different valence states, vanadium ions are the most studied^7^. The rate of electron hopping between ions is facilitated when vanadium ions are present in either of the two valence states, viz., ${{\text{V}}}^{4+}$ or ${{\text{V}}}^{5+}$, which eventually results in an increase in electrical conductivity^8^. Vanadium-containing glasses have numerous uses in memory, solid state batteries, and switching devices^9^. Because of their wide radial distribution of outer d-electron orbital functions and their extreme sensitivity to changes in the surrounding cations, transition metal ions are often employed in glass structure probes^10^. Applications for glasses containing V_2_O_5_ and B_2_O_3_ can be found in optoelectronics and memory switching devices^11^.

High refractive indexes, high polarizability, high density, high valence cation, strong nonlinear optical susceptibility, and excellent infrared transmission are characteristics of bismuth oxide^12^. Bi_2_O_3_ is not regarded as network-forming because ${{\text{Bi}}}^{3+}$ ion has low field strength. However, a wide range of compositions may result in a high probability of glass formation when Bi_2_O_3_ and B_2_O_3_ are mixed to create bismuth-borate glasses.

When bismuth is in its monoclinic form, the octahedral adjustment of six oxygen atoms is positioned at an ionic radius of 2.14 to 2.29 Å, with three oxygen atoms being much closer, at around 2.29 Å.

The states of bismuth ions are ${{\text{Bi}}}^{+}$,${{\text{Bi}}}^{3+}$, ${{\text{Bi}}}^{4+}$ and ${{\text{Bi}}}^{5+}.$ Compared to other Bi cations, ${{\text{Bi}}}^{3+}$ the cation exhibits more stability, which further qualifies the glass as a non-linear optical or photonic material with a high non-linear optical susceptibility^13^. Because of this, these glasses are important materials for low loss optical fibre^14^, processing devices^15^, and radiation shields^16^. Additionally, the glass matrix’s incorporation of zinc oxide reduces the band gap and raises the refractive index. Zinc oxide fills the gaps in the glass matrix to act as both a network former and a network modifier^17^. Numerous researchers are studying different characteristics of borate glasses with different oxides^18–22^.

The objective of the current study is therefore to study the influence of Bi_2_O_3_ content on the physical, structural, optical, and electrical properties of 10K_2_O–10ZnO–55B_2_O_3_–(25–x) V_2_O_5_–xBi_2_O_3_ glass systems. The concentration of Bi_2_O_3_ is limited from 4 to 10 mol% in the present glass system because the glass formation gets harder and becomes crystalline.

### Experimental details

#### The preparation of samples

**Figure 1:**
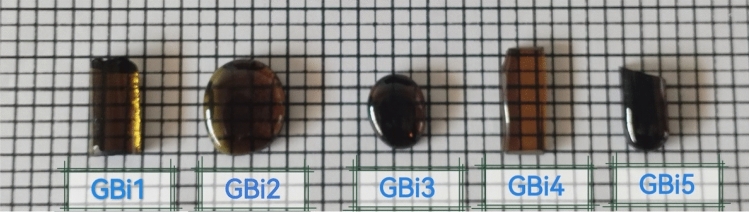
Images of all the investigated glass samples.

Five glass samples GBi1, GBi2, GBi3, GBi4 and GBi5 having chemical composition 10K_2_O–10ZnO–55 B_2_O_3_–(25–x) V_2_O_5_–x Bi_2_O_3_ (where x varies from 4 to 10 mol%) were fabricated by using melt-quenching technique. The chemical compositions of different glass samples fabricated along with their labels are listed in Table 1. Highly pure analytical grade K_2_CO_3_, ZnO, H_3_BO_3_, V_2_O_5_ and Bi_2_O_3_ chemicals were used as starting materials. The well ground mixture of chemicals in appropriate weight ratios were taken in porcelain crucibles and melted in an electrical muffle furnace at temperature 1250 $\mathrm{^\circ{\rm C} }$. The melt was poured on a preheated stainless steel plate. The quenched samples were annealed at 450 $\mathrm{^\circ{\rm C} }$ for 3 h and then left in the furnace to cool down to room temperature to reduce the internal stress. These samples were cut and then will undergo polishing and grinding process to analyze the glass samples for its characteristics. Images of the glass samples are displayed in Fig. 1.

**Table 1:** Chemical composition, density and molar volume of the prepared glasses.

Sample ID	Chemical composition (mol%)					Density (g/cm^3^)	Molar volume (mol/cm^3^)	Glass forming
	K_2_O	ZnO	B_2_O_3_	V_2_O_5_	Bi_2_O_3_			
GBi0	10	10	55	25	–	–	–	Crystallized
GBi1	10	10	55	21	4	2.7878	40.4196	Glass
GBi2	10	10	55	20	5	2.8789	40.1273	Glass
GBi3	10	10	55	17.5	7.5	3.1418	39.0299	Glass
GBi4	10	10	55	16	9	3.2738	38.7579	Glass
GBi5	10	10	55	15	10	3.3617	38.5895	Glass
GBi6	10	10	55	–	25	–	–	Crystallized

#### Samples characterization

To confirm the amorphous nature of the prepared samples, X-ray patterns of the glass samples have been recorded by using a Rigaku Table-Top X-ray diffractometer with source Cu Ka radiation in the 2θ range 10°–80° at a scanning rate of 10 min.

The tightness, rigidity and structural changes of the obtained glass samples can be investigated through the measurement of the density (*ρ*) of glasses was measured at room temperature based on Archimedes methods and can be calculated by the following equation:

1 $$p\, = \,\frac{{m_{1} }}{{m_{1} - m_{2} }} \times p_{0}$$

Where ${m}_{1}$ is the weight of the sample in the air, ${m}_{2}$ is the weight in distilled water, respectively, and ${\rho }_{0}$ is the density of water (= 0.9989 g/cm^3^). The value of molar volume (${V}_{m}$) is related to the compaction of the glass network and can be calculated as follows:

2 $$V_{m} = \sum {} x_{i} \,M_{i} \,/\,{\uprho }$$

Where ${x}_{i}$ is the molar fraction, ${M}_{i}$ is the molecular weight of component $\left\{i\right\}$.

Fourier transform infrared spectroscopy (FTIR) spectra of the glasses were recorded in the wavenumber range 400–4000 cm^−1^ using (Jasco-6100, Japan). The measurements were calculated using the KBr pellet technique.

Optical absorption measurements of the prepared samples were performed using a Cary series UV/Vis-spectrophotometer at room temperature in the range of 200–1100 nm.

The conductivity of the prepared samples was measured using Novocontrol Technologies, GmbH& Co. KG, high-resolution alpha analyser (0.1–20 MHz) in the temperature range 25–200 °C and stabilized with an accuracy of more than 0.1o Cusing Quattro temperature controllers employing pure nitrogen gas as the heating agent. The cell used was calibrated using standard materials (air, Trolitul and glass) with different thicknesses ranging from 1 mm up to 7 mm at 10 kHz with an LCR meter. Calibration curves were tested with two Teflon samples of different thicknesses, and it was found that the error in εʹ amounts to ± 2% and that the standard deviation amounts to 0.04.

## Results and discussion

### X-ray diffraction

**Figure 2:**
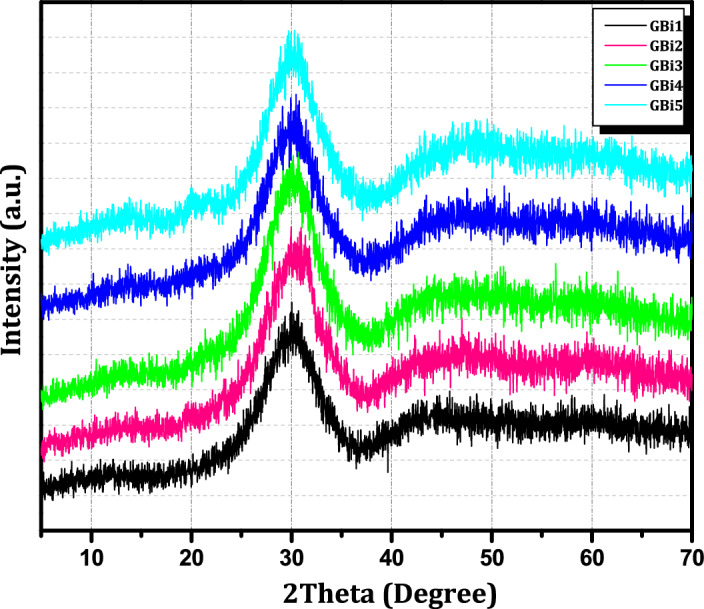
XRD patterns for all the compositions of glass samples (GBi1 –GBi5).

Using X-ray diffraction data, the glassy phase of the manufactured glass systems is displayed in Fig. 2. The XRD analysis demonstrated the complete amorphous nature of each glass sample and the absence of a uniform atom arrangement that would have been present in a crystal case. Due to variations in interatomic distance, glasses exhibit a wide range peak, as seen by the emergence of a broad hump in the range of 20$^\circ$–40$^\circ$ for glass composition^23,24^. All of the glass samples are in the amorphous or non-crystalline phase, as demonstrated by this behavior. The ability of the borate glass networks to form glass was improved, and bulk glass samples were more transparent and clear as a result of the addition of Bi_2_O_3_^23–25^.

### Density and molar volume characterization

**Figure 3:**
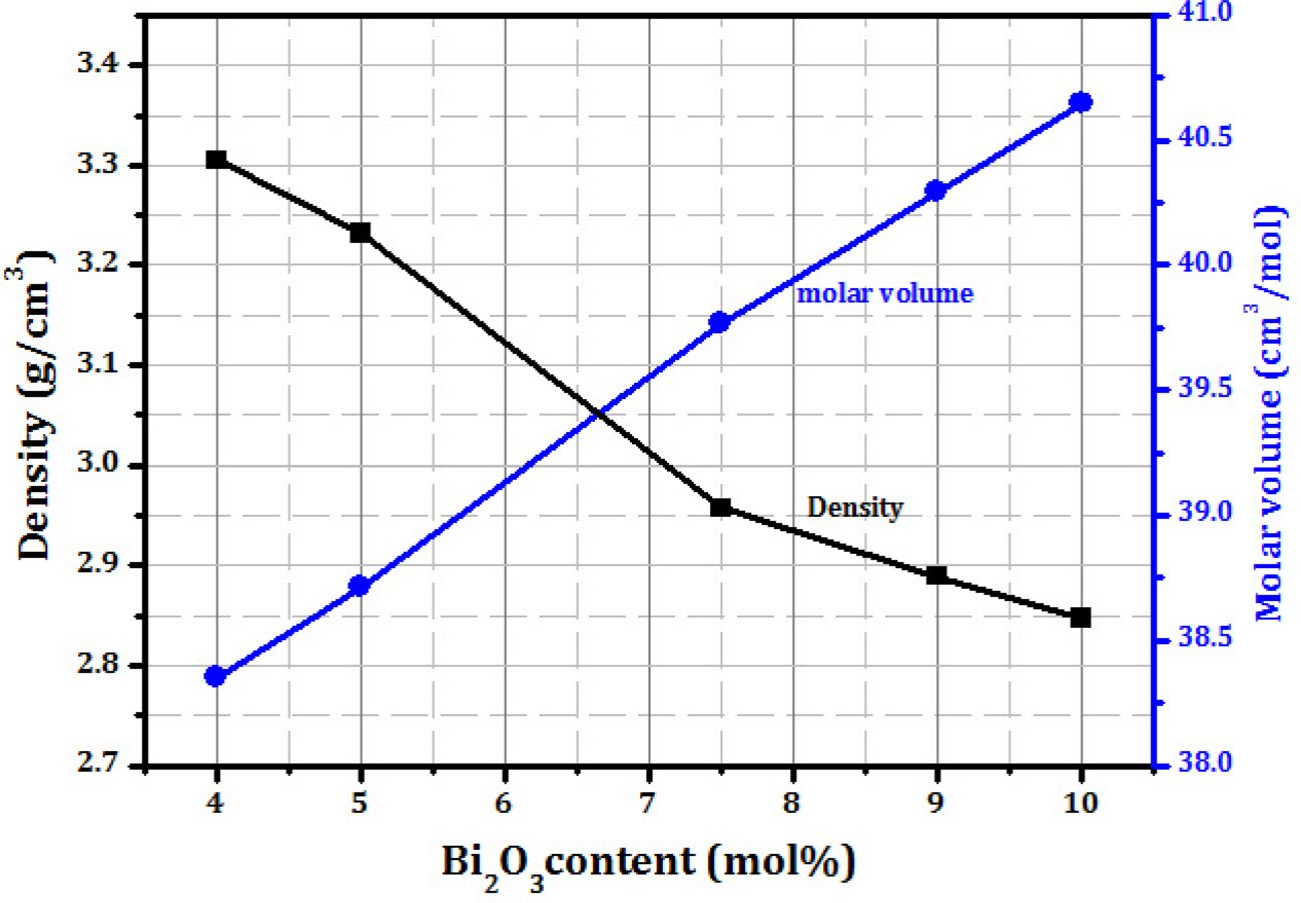
Density and molar volume as a function of Bi_2_O_3_ content in glass samples.

Table 1 lists the density values (ρ) for each produced glass sample that was obtained. With the addition of bismuth oxide, the density values exhibit an increasing tendency in the following order: GBi1 < GBi2 < GBi3 < GBi4 < GBi5. This is because Bi_2_O_3_ has a high molecular weight and density(465.96 g/mol, 8.9 g cm^−3^) compared to V_2_O_5_ (181.88 g/mol, 3.36 g cm^−3^), the density increased from 2.7878 to 3.3617 g cm^−3^ as expected with the substitution of V_2_O_5_ with Bi_2_O_3_^26^. In the meantime, the molar volume value and the density measurement typically behave in opposite directions. In contrast to the observed density, this investigation showed that the molar volume (${V}_{m}$) decreases in the order GBi1 > GBi2 > GBi3 > GBi4 > GBi5. Figure 3 shows the molar volume and experimental density of the produced glasses as a function of the Bi_2_O_3_ content. Finally, it's possible that Bi_2_O_3_ functions as a network modifier, forming non-bridging oxygen’s (NBO’s) atoms that alter the borate glass's structural composition. The concentration of non-bridging oxygens in the glass network increases when the bismuth oxide replaces the vanadium oxide, converting the [BO_3_] structural units into [BO_4_] structural units^27–29^.

### Fourier transforms infrared spectroscopy studies (FTIR)

**Figure 4:**
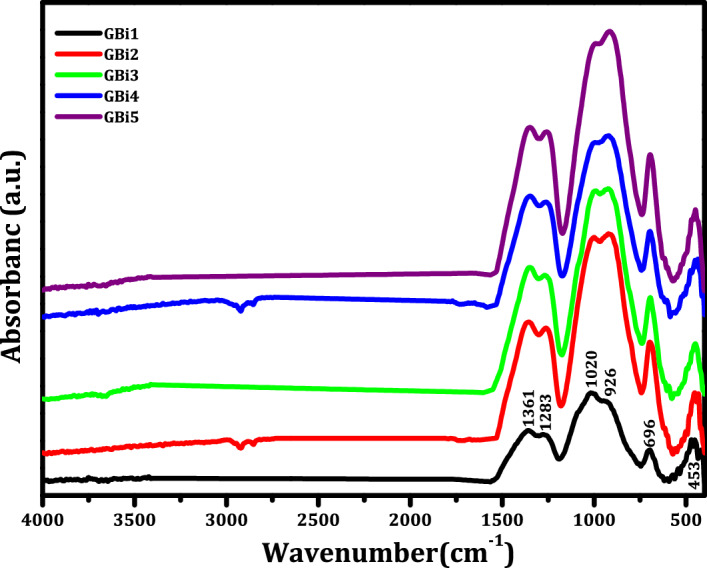
FTIR spectra of present glasses as a function of Bi_2_O_3_ mol%.

In order to investigate how the interactions between the different atoms in the samples affected their structure, infrared spectroscopy was employed. Table 2 displays the band positions and peak assignments of the FTIR spectra that were obtained for each produced glass within a 4000–400 cm^−1^ spectral range, as depicted in Fig. 4.

**Table 2:** Infrared wavenumber and assignments of vibrational modes of 10K_2_O–10ZnO–55 B_2_O_3_–(25–x) V_2_O_5_– x Bi_2_O_3_ glass systems.

Wavenumber (cm^−1^)	IR band assignments
470	Bi–O bending vibrations in BiO_6_ and/or BiO_3_ units / Bending of BO_4_ units
542	Bending modes of V–O–V bonds and/or Bi–O / Bi–O–Bi stretching vibrations of [BiO_6_] octahedral structural units
699	B–O–B bending vibrations BO_3_ groups in borate network
930	Stretching vibrations of tetrahedral BO_4_ units / V=O vibration of [VO_5_] vanadium group
1005	B–O stretching vibrations of tetragonal [BO_4_] units in tri-, tetra- and penta- borate groups
1267	B–O stretching vibrations of trigonal [BO_3_] units from boroxol rings with non-bridging oxygen atoms
1378	Asymmetric B–O stretching vibrations of trigonal [BO_3_]^3−^ units in meta-, pyro-, and ortho-borate groups

The glass network contains a variety of links and vibrational modes. Three basic groups are present in the vibrational modes of borate glass: (600–800) cm^−1^, (800–1200) cm^−1^, and (1200–1600) cm^−1^. In triangular BO_3_ structural units, the bending vibrations of the B–O–B and the stretching vibrations of the B–O bond are often associated with the first and third absorption regions. However, the second region is caused by the stretching vibrations of the tetrahedral BO_4_ structural units^30^.

The well-defined peaks in the infrared spectra located at 470 cm^−1^ are due to the vibration in the local symmetry of highly distorted BiO_6_ polyhedral units and/or BiO_3_ units and/or bending of BO_4_ units^31,32^. Another IR peak at 542 cm^−1^ may be attributed to Bi–O and Bi–O–Bi stretching vibrations of [BiO_6_] octahedral structural units and/or bending vibration of the V–O–V bond^33,34^. The BiO_3_ polyhedra vibration band does not show in the IR absorption^35^. Therefore, the bismuth structure that exists in the glasses is solely attributed to the [BiO_6_] octahedral units.

In the borate network, the absorption band detected at 699 cm^−1^ is connected with B–O–B bending vibrations of BO_3_ groups^36,37^. The combination of the V=O vibration of the [VO_5_] vanadium group and the stretching vibration of the B–O bond in the [BO_4_] tetrahedral units is responsible for the absorption peak at 930 cm^−1^^38,39^.

This band obviously changes towards longer wave numbers as a result of the [VO_4_] groups becoming [VO_5_] groups^40^. The absorption band observed at 1005 cm^−1^ is related to B–O stretching vibrations of tetragonal [BO_4_] units in tri-, tetra- and penta-borate groups^41,42^. Absorption peaks at around 1267 cm^−1^ are produced by the B–O stretching vibrations of trigonal [BO_3_] units from boroxol rings containing non-bridging oxygen atoms^43,44^.

Trigonal [BO_3_] units in the meta-, pyro-, and ortho-borate groups have asymmetric B–O stretching vibrations, which are linked to the absorption band found at 1378 cm^−1^^45,46^.

However, the absence of the distinctive 800 cm^−1^ boroxol ring band, which is typically present for borate networks, suggests that there are no boroxol rings in the borate network. As a result, BO_3_ and BO_4_ structural groups make up the majority of the glass samples^47^. In these compositions, bismuth is expected to function as a network modifier. The BO_3_ triangle's structure, however, changed as the content of Bi_2_O_3_ increased to produce the BO_4_ tetrahedral, which is close to the energy needed to break B–O–B bridges and form non-bridging oxygen and forms different kinds of structural units^48^.

### Optical properties

#### UV–visible analysis

**Figure 5:**
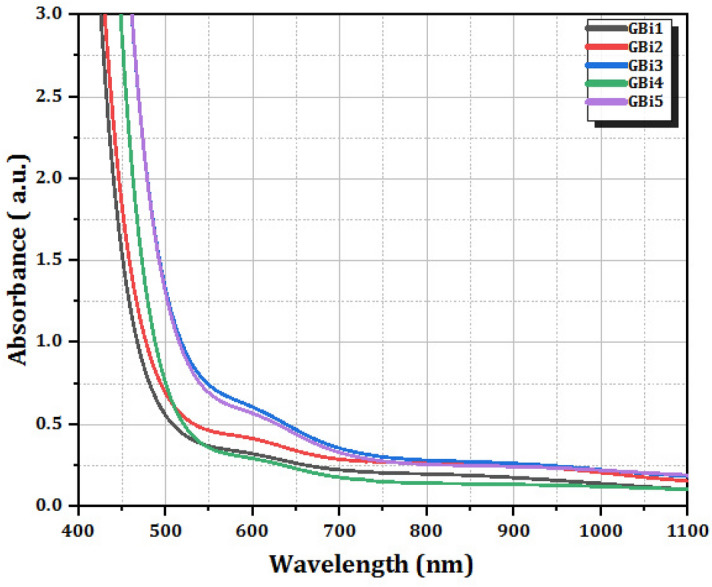
Optical absorption spectra of glass series.

One effective method for examining the electrical structures of amorphous semiconductors is the examination of optical absorption spectra^49^. The UV–visible absorption spectra of the glass samples with different Bi_2_O_3_ contents are shown in Fig. 5 in the wavelength range of 200–1100 nm. The bandgap, oxygen deprivation, surface roughness, and impurity centres are some of the variables that affect absorbance^50^. A straight line was drawn to determine the cut-off wavelength *(*${\lambda }_{C}$), and after the line crossed the wavelength axis, the cut-off wavelength was selected^51^.

The studied samples exhibited an increase in absorbance in the visible region upon increasing the Bi_2_O_3_ content. Tetravalent ${{\text{V}}}^{3+}$ ions are exactly attributed to the absorption band at 597 nm. It is believed that ${{\text{V}}}^{3+}$ ensures three spin-allowed absorption transitions in tetrahedral and octahedral coordination. In oxide glasses, ${{\text{V}}}^{3+}$ cause absorption bands that represent the transitions from ^3^T_1g_ (F) to ^3^T_2g_ and ^3^T_1g_ (P) states, respectively^52^.

It has been observed that as the amount of Bi_2_O_3_ in borate glass structures increases, the optical absorption cut-off wavelength shifts from a lower wavelength to a higher wavelength value. As indicated in Table 3, the optical cut-off wavelength of the glasses under study has been moved from 472 to 521 nm. Because of the gradual formation of NBOs in the glass networks, the altered behavior of the absorption cut-off wavelength can be linked to reduced glass structure stiffness. Glass networks are degraded because non-bridging oxygen electron bonding is less tightly bound than bridging oxygen bonding^53^. Consequently, a decrease in the optical band gap energy would result from the breaking down of the BO's bond and a shift in the absorption edge to a longer wavelength.

**Table 3:** Cutoff wavelength (λ_c_), optical band gap energy *E*_opt_ (direct), optical band gap energy *E*_opt_ (indirect) and Urbach energy (∆E) of the prepared glasses.

Sample ID	Cut-off wavelength(λ_c_), nm	*E*_opt_ (direct), eV	*E*_opt_ (indirect), eV	∆E, eV
GBi1	472	2.7984	2.3627	0.743
GBi2	488	2.7036	2.3139	0.814
GBi3	537	2.5444	2.2127	0.830
GBi4	502	2.6087	2.2573	0.526
GBi5	521	2.5230	2.1634	0.687

Glass’s band gap energy is determined by analyzing its UV absorption edge. To get the absorption coefficient α (ν) close to the spectrum edge, use Eq. (3)^54^:

3 $${\upalpha }\left( {\text{v}} \right)\,{ = }\,{2}{\text{.303}}\,{\text{A/d}}$$

where d represents the glass sample's thickness and A its absorbance. Davis and Mott^55^ report that optical absorption of amorphous materials occurs above the exponential tail with a larger value of α (ν), following a power law expressed by Eq. (4):

4 $${\upalpha }\left( {\text{v}} \right){\text{hv}}\,{ = }\,{\text{B}}\left( {{\text{hv}}\, - \,{\text{E}}_{{{\text{opt}}}} } \right)^{{\text{n}}}$$

where hυ is the incident photon energy, α (ν) is the optical absorption coefficient, B is constant, n is the index that is defined by the type of electronic transitions that occur during the absorption process, and E_opt_ is the optical band gap energy between the valence band and the conduction band. The value of *n* can be either *n* = 1/2 and *n* = 3/2 for direct allowed and direct forbidden transitions or *n* = 2 and *n* = 3 for indirect allowed and indirect forbidden transition.

**Figure 6:**
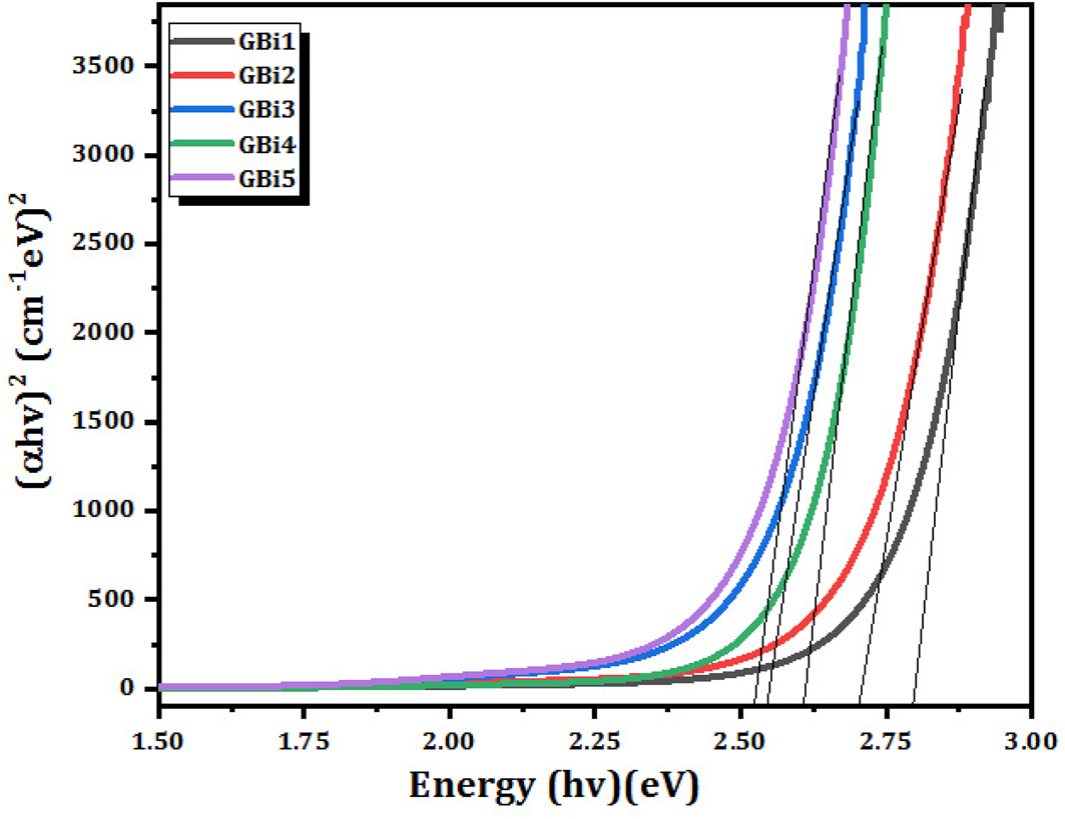
(α*h*ν)^2^ as function of photon energy *h*ν of glass samples (direct transition).

**Figure 7:**
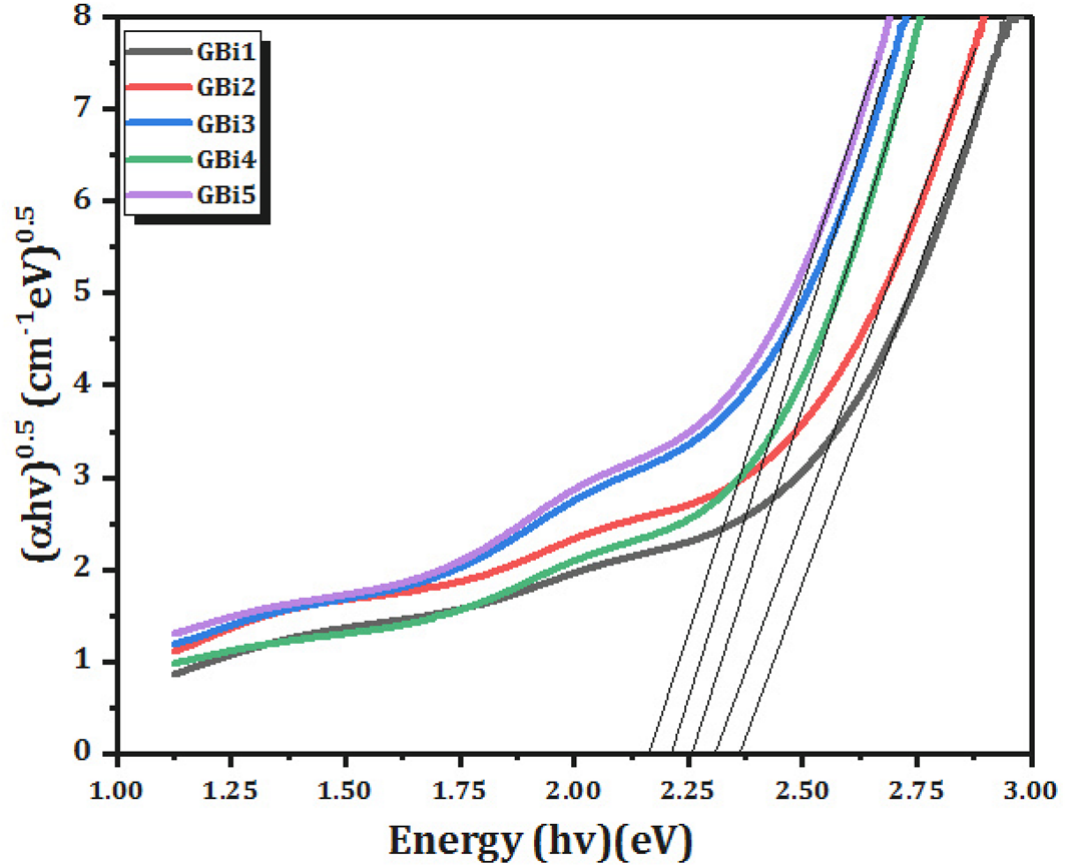
(α*h*ν)^0.5^ as function of photon energy *h*ν of glass samples (indirect transition).

Plotting (α*h*υ)^0.5^ and (α*h*υ)^2^ vs the photon energy (hυ), Eq. (4) was used in this work to calculate the indirect and direct allowable optical energy band gap, or *E*_*opt*_, for glass samples. It is possible to calculate the optical energy band gap by extrapolating the linear portion of the observed curves to lower energy. Table 3 provides a summary of the relationship between *E*_*opt*_ values and Bi_2_O_3_content for both direct and indirect transitions, as illustrated in Figs. 6 and 7, respectively.

In borate glass systems, the optical energy gap (*E*_*opt*_) takes values between 2.7984 and 2.5230 eV in the case of a direct transition (Fig. 6), and ranges from 2.3627 to 2.1643 eV for an indirect transition (Fig. 7). Essentially, the changes in structure within the networks of borate glass are causing the optical band gap to decrease, as previously determined by researchers^56,57^.

By producing a concentration of NBO, the replacement of Bi_2_O_3_, which acts as a glass modifier, would disrupt the regular structure of borate glass networks, making the glass structure more random^58^. However, it is also thought that because Bi_2_O_3_ elements are highly polarizable and easily deformed by cations, as cation concentrations increase, the bridging oxygen will form a bond with Bi^3+^ ions and the glass networks will gradually break down^59^. The concentration of non-bridging oxygens (NBOs) is often increased by an increase in the network modifier concentration, and states originating from NBOs are easier to excite than ones originating from bridging oxygen atoms. As a result, the optical band gap reduces^60^.

An essential parameter that indicates the degree of disorder in amorphous materials is the Urbach energy (∆E). Following the empirical Urbach rule, the relationship between Urbach energy (∆E) and absorption coefficient α (v) is given^61^:

5 $${\upalpha }\left( {\text{v}} \right)\, = \,{\text{B}}\,\exp \left( {hv/\Delta E} \right)$$

where B is constant and ∆E is Urbach energy, which corresponds to the width of the band tails of localized state. The relation can be rewritten as:

6 $${\text{In}}\,{\upalpha }\left( {\text{v}} \right)\, = \,hv/\Delta E + cons\tan t$$

Urbach energy values for the glass samples are listed in Table 3, and the values of ΔE were computed by taking the reciprocals of the slopes of the linear portion in the low photon energy region of ln(α) versus $h\nu$ plot (not shown). Also, the tails are affected by the disorder level and the structure of the sample^62^.

#### Some other optical parameters

The Dimitrov-Sakka relation can be used to calculate the refractive index from optical band gap energy^63^.

7 $$\left(\frac{{{\text{n}}}^{2}-1}{{{\text{n}}}^{2}+2}\right)=1-\sqrt{\frac{{{\text{E}}}_{{\text{opt}}}}{20}}$$

where ${{\text{E}}}_{opt}$ is optical band gap energy and n is the refractive index. Because glasses are amorphous by nature, most indirect transitions occur as a result of the electrons' undefined momentum. For this reason, the refractive index is only determined via indirect bandgap energy. Table 4 shows that there is a slight increase in refractive index with increasing Bi_2_O_3_ content, ranging from 2.5939 to 2.6686. Since non-bridging oxygens are more polarizable than bridging oxygens, this kind of increase may be explained by an increasing amount of these oxygens. The glass structure is changed by the non-bridging oxygens, making the molecular packing denser. The reason for this denser packing is that more network modifiers are occupied at intestinal sites. Given that a glass system's refractive index and density are closely correlated, a glass with a higher density will also have a higher refractive index^64^.

**Table 4:** Optical parameters of the studied glasses.

Sample ID	Refractive Index, (n)	Molar refraction, (${R}_{m}$) (cm^3^/mol)	Metallization criterion, (*M*)	Optical electronegativity, (χ)	Dielectric constant, (ε)	Optical dielectric constants, (${\upvarepsilon }_{{\text{opt}}}$)	Optical polarizability, (${\mathrm{\alpha }}_{0}$)
GBi1	2.5939	26.5270	0.3437	0.6351	6.7283	5.7283	2.9284
GBi2	2.6115	26.4784	0.3401	0.6220	6.8199	5.8199	2.9402
GBi3	2.6494	26.0478	0.3326	0.5948	7.0193	6.0193	2.9647
GBi4	2.6325	25.7374	0.3359	0.6068	6.9300	5.9300	2.9539
GBi5	2.6686	25.8976	0.3289	0.5815	7.1214	6.1214	2.9766

Lorentz–Lorentz provides the correlation between molar refractivity ($${R}_{m })$$ and molar volume^65^.

8 $${{\text{R}}}_{\mathrm{m }=}\left(\frac{{{\text{n}}}^{2}-1}{{{\text{n}}}^{2}+2}\right){{\text{V}}}_{{\text{m}}}$$

Molar refractivity values have opposite trends in the optical energy and its values decrease from 26.5270 to 25.8976. Also, molar refractivity is essential for understanding and predicting a material's conduction behavior.

Glass is determined to be metallic or insulator by calculating the metallization criterion (*M*), which takes into consideration the ratio of ${R}_{m}$/${V}_{m}$ and can be stated as follows^66^.

9 $${\text{M}}\, = \left( {1 - \frac{{R_{m} }}{{V_{m} }}} \right)$$

Herzfeld's metallization theory^67^ specifies the criteria for classifying solids as either non-metallic (${R}_{m}$/${V}_{m}$ $<$ 1) or metallic (${R}_{m}$/${V}_{m}$ $\ge$ 1) depending on their characteristics. The calculated values of *M* are listed in Table 4. If metallization criterion reaches to 1 means the materials are becoming insulators, instead if it reaches to 0 the materials becoming conductors^66^. The glasses under investigation show a greater tendency towards metallization as determined by the criterion of small metallization (${R}_{m}$*/*${V}_{m} is$ large). The obtained optical band gap energy measurements are in agreement with the results of the metallization criteria^68^.

The refractive index was used to compute the dielectric constants and optical dielectric constants of the prepared samples, as indicated by the following expressions:

10 $$\varepsilon ={n}^{2}$$

11 $$\varepsilon_{{{\text{opt}}}} = \varepsilon - 1$$

The empirical formulas were used to calculate characteristics like electronegativity (χ) and optical polarizability (${\alpha }_{0}$)^69^.

12 $$X\, = \,0.2688\,*{\text{E}}_{{{\text{opt}}}}$$

13 $${\upalpha }_{{0}} = - 0.9\,x\, + 3.5$$

One property of oxide glasses called electronegativity shows how strongly an ion may bind electrons. There is weaker bonding across ion networks as a result of the ions' reduced electronegativity, which causes them to attract adjacent oxide ions less strongly^68^. The values of the optical polarizability of the prepared glasses increased from 2.9284 to 2.9766 due to a decrease in electronegativity (χ). These parameter values are listed in Table 4.

### Electrical properties

#### Ac-conductivity

**Figure 8:**
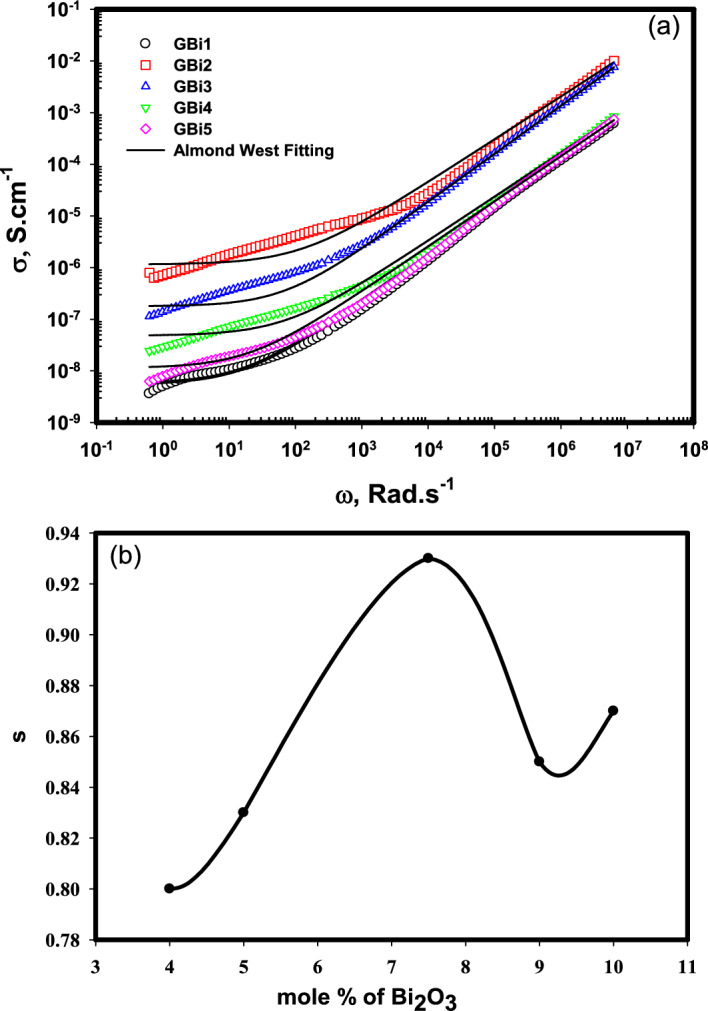
(**a**) The frequency dependence of ac conductivity (*σ*_*ac*_) for glasses (GBi1-GBi5), (**b**) The Bi_2_O_3_ content dependence of exponent factor s.

Studying the behavior of alternating-current conductivity (*σ*_ac_) of the prepared glasses is very important to determine the extent of the glasses to conduction under the effect of an electric field. Ac-conductivity of different glass compositions GBi1, GBi2, GBi3, GBi4, and GBi5 over the frequency region 10^−1^–10^6^ Hz at room temperature are shown in Fig. 8a.

The frequency (*f*) dependence of Ac-conductivity (*σ*_*ac*_) is usually expressed by the following Jonscher relation Eq. (14) and Almond-West formalism Eq. (15)^70,71^:

14 $${\sigma }_{ac}={\sigma }_{dc}+A{\omega }^{s}$$

15 $${\sigma }_{total}\left(\omega \right)= {\sigma }_{dc}[1+\frac{\omega }{{\omega }_{H}}{]}^{s}$$

Where ω represents the frequency and equals to *2πf* and called as the angular frequency, *s* is the frequency-exponent which have values (0 < *s* ≤ 1), ${\sigma }_{dc}$ is the dc-conductivity, A is a constant, and ω_H_ is the crossover frequency which indicates the frequency at which the frequency-independent region separates from the dispersion conductivity region. Figure 8a shows a delay in the values of *σ*_*ac*_ with decreasing the frequency due to the presence of free charge carriers at the electrode surface that causes electrode polarization (EP)^72^. At very low frequency values, the conductivity attains nearly constant value which is attributed to the dc-conductivity (*σ*_*dc*_) which originated from the jumping of ions to the adjacent vacant site or from the diffusion of the ionic charge carriers^73^. In our samples, the reason of this conduction is mainly due to the electron transfer through V^4+^-O-V^5+^^74^. The data of Fig. 8a was non-linearly fitted by Almond-West formalism and the parameters of the fitting were listed in Table 5. As listed in the table, the values of *σ*_*dc*_ are ranging from 10^−6^–10^−9^ S cm^−1^, which in agreement with the behavior of glasses contains transition metal where the electronic conductivity of these glasses is predominant^74^. The estimated values of s are used to define the mechanism by which the charge transferred^75–77^. As shown from Table 5, s < 1 indicates that the conduction occurs through hopping of charges between two potential barrier sites^78,79^.

**Table 5:** DC conductivity (σ_dc_), crossover frequency (ω_H_) and frequency factor (s) of the glasses synthesized in the system.

Sample ID	σ_dc_, S cm^−1^	ω_H_	s
GBi1	5.6 × 10^−9^	2.6	0.80
GBi2	1.1 × 10^−6^	19.2	0.83
GBi3	1.8 × 10^−7^	10.7	0.93
GBi4	8.1 × 10^−8^	11.2	0.85
GBi5	1.1 × 10^−8^	3.2	0.87

The conductivity increases with increasing the frequency which indicates the semiconductor character of the examined samples, it also increases as the amount of Bi_2_O_3_ in borate glass structures increases due to the presence of two oxidation state of Bi^3+^ and Bi^5+^ that share in the jumping process where one of them plays as a donor and the other as acceptor, respectively^80^. The presence of Bi in the structure of glass containing transition metal (V) can lead to decrease the bond distance in V–O–V that leads to increase in the V^5+^/V^4+^ ratio^81–83^. In addition to the production of the tetrahedral BO_4_ increases by increasing the Bi_2_O_3_ content that results in increasing the donner Bi^3+^ and the formation of non-bridging oxygen as discussed in the IR results. Also, the presence of shift in the wave number of [VO_4_] towards longer wave numbers indicates its change to the trigonal bipyramids [VO_5_] groups^40^.

It is worth to mention that the values of conductivity for all the samples are ranging from ~ 10^−8^ at low frequency to ~ 10^−2^ at high frequency that specifies the semiconductor character of the samples. The s values were drawn as a function of the Bi_2_O_3_ content in borate glass as shown in Fig. 8b, where s increases with increasing Bi content till 7.5% then decreases but still its value > GBi1, this behavior is due to the formation of NBO with the increase in Bi_2_O_3_ content, while the decrease of s value for Bi_2_O_3_ content > 7.5% may be because of the disturbance in the NBO in glasses^84^.

#### The permittivity and dielectric loss

To identify the stored energy in the studied glasses under the effect of electric current, the real part of the dielectric constant (permittivity) (*ε'*) was measured.

**Figure 9:**
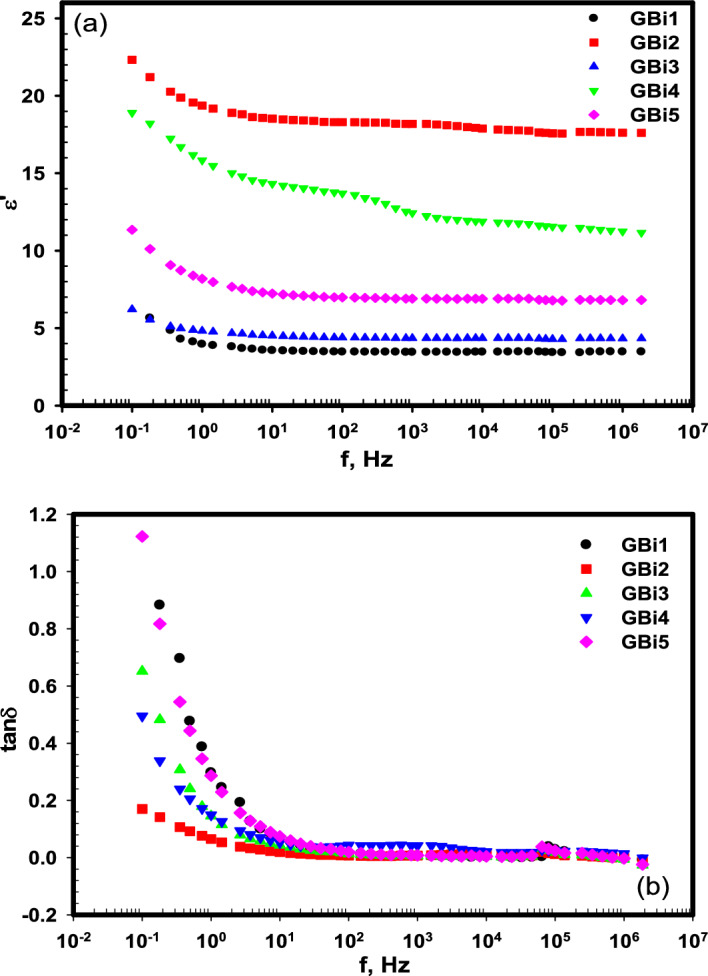
The frequency dependence of (**a**) dielectric constant (εʹ), (**b**) dielectric loss (tanδ) for the glass samples (GBi1-GBi5).

The frequency dependence of *ε'* for the studied glasses is shown in Fig. 9a, it is noted that *ε'* is affected by both the composition of the glasses and the frequency of the electric field, it increases with increasing the Bi_2_O_3_ content due to the increase of both polarizability of glass and nonbridging oxygen (NBO)^85^. For all the studied glass compositions, it attains high value at low frequency due to the presence of different kinds of polarization such as the space charge and the dipole polarizations^86^. As the glass is amorphous, therefore there is a defect in its bulk interface that results in transferring the space charges at the presence of an electric field. Therefore, the predominant polarization of glass in low frequency values is space charge polarization^87,88^. Then, a gradual decrease in *ε'* was observed with raising the frequency due to the dielectric relaxation phenomenon that happened because of the instability of the localization of charge carrier localization under the electric field effect^89^. At frequency > 10^4^ Hz, unchanged *ε'* value is achieved indicating the independence of *ε'* on the electric field.

To recognize the dissipated of energy in the studied glasses, the tangent loss (*tanδ*) was calculated from the dielectric loss (*ε''*) and *ε'* as the following equation:

16 $$tan\delta \left(\omega \right)=\frac{{\varepsilon ^{\prime}}{^{\prime}}}{{\varepsilon ^{\prime}}}$$

Figure 9(b) shows the change in *tanδ* with frequency for the studied glass samples, which looks like the change of *ε'* with frequency. At low frequency, *tanδ* have high values that decreases gradually as the frequency increases till 100 Hz, after that it reaches nearly constant, then a relaxation peak is observed at nearly 31.6 kHz which may be dipolar relaxation. It was also noted that *tanδ* decreased with increasing Bi_2_O_3_ content and GBi2 achieves the highest value of dielectric constant and the lowest value of dielectric loss.

#### Impedance measurements

**Figure 10:**
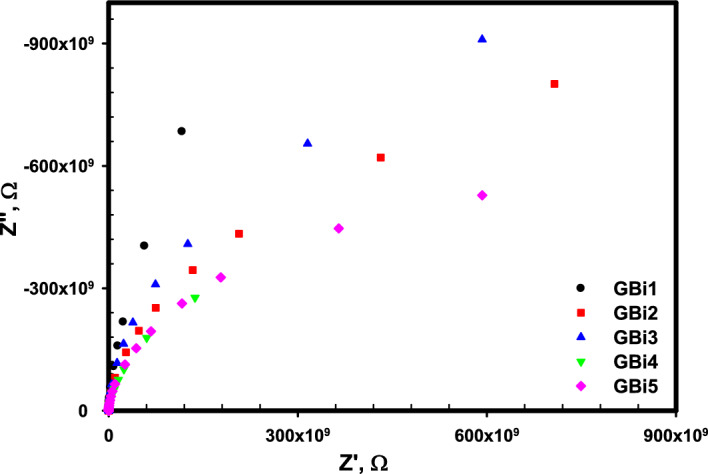
Nyquist plots for the glass samples (GBi1-GBi5) at room temperature.

The measurement of impedance for the studied samples is represented by the Nyquist plots that give how can the real part of impedance changed with the imaginary parts at room temperature are shown in Fig. 10. This relation can help in understanding the role of the microscopic elements of the material, such as the grain, electrode effect, and relaxation process^90^. Inclined lines tend to bend at the x-axis to shape as semi-circles that interrelated to the capacitance and resistance of the bulk were observed in the figure. The angle by which the line is inclined decreases with increasing the Bi_2_O_3_ content that means the semicircle radius reduces that indicates the increase in the conductivity of the bulk with Bi_2_O_3_ concentration rising. This behavior is coincidence with the conductivity measurements. As the semicircle is asymmetric (depressed), therefore a deviation from Debye relaxation occurs that may be due to different factors such as the dipole groups formation, a defect in the atomic distribution and formation of nonpolar clusters^91^. Grain orientation, defect in the atomic distribution of the grain boundaries and the stress strain in the glass materials are from the factors that causes this nonideal behavior. However, the presence of one semicircle reveals that the glass system conducting behavior comes mainly from the grains rather than the grain boundaries^92^.

#### The electrical modulus analysis

**Figure 11:**
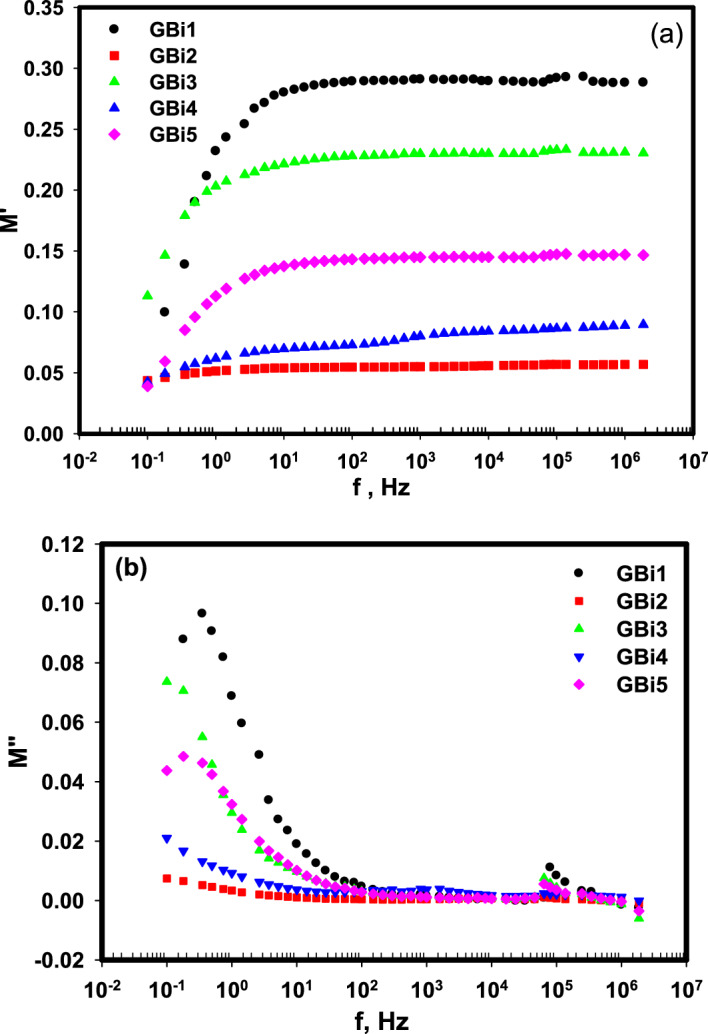
The frequency dependence of (**a**) M′ (real part), (**b**) M″ (imaginary part) of electric modulus for the glass samples (GBi1-GBi5).

To investigate the relaxation process and to understand the response of the bulk, the variation of the real part of electric modulus Mʹ, and its imaginary part M″ with the frequency was investigated as in Fig. 11a,b. Low values of M' were observed at low frequency, then a gradual enhancement in Mʹ occurred and went to higher values with rising the frequency, then accomplished maximum value at *f* > 20 Hz. This behavior demonstrates the dispersion of the relaxation processes along all the studied frequency range^93^. The mobility of the charge carriers is the reason for the increase in M′, where the effect of the electric field on their mobility is restricted^94^.

The behavior of changing M″ with frequency (Fig. 11b) shows an indication of a peak at low frequencies and its position changed to lower values of frequency as the Bi_2_O_3_ content increases which directs the involvement of dc-conductivity^95,96^. Another peak with lower height is observed at high frequency and its height reduces with high Bi_2_O_3_ content (inset Fig. 11b). Control in the charge carriers occurred between the two peaks.

## Conclusions

In this study, potassium–zinc–borovanadate glass containing different concentrations of bismuth oxide was examined with respect to physical, structural, optical, and electrical properties. To achieve this, a new glass series with composition 10K_2_O–10ZnO–55B_2_O_3_– (25–x) V_2_O_5_– x Bi_2_O_3_ (x: 0, 4, 5, 7.5, 9, 10 mol%) was synthesized using the traditional melt-quenching route. A broad peak was identified by analyzing the XRD pattern of the samples, indicating that they are non-crystalline or amorphous in nature. Various physical characteristics were identified, including density and molar volume. The density of the glass samples increases with an increase in the content of Bi_2_O_3_ and causes a corresponding decrease in the molar volume. The glasses' UV–VIS spectra demonstrate that the addition of Bi_2_O_3_ caused the absorption edge to move towards a higher wavelength. Additionally, the glasses' direct and indirect optical band gaps showed a tendency to decrease upon the addition of Bi_2_O_3_ and the enhancement in Urbach energies (ΔE) of glasses. The development of non-bridging oxygen in the glass system is responsible for this, due to an increase in BiO_6_ octahedral units, as observed from FTIR analysis. The metallization criteria (M) indicate that the glasses have a greater tendency towards metallization. Both the conductivity and the dielectric constant increase with the rise in Bi_2_O_3_ content due to increasing the polarizability and NBO; however, the dielectric loss and the impedance reduce. The values of conductivity for the studied glasses ranged from ~ 10^−8^ to ~ 10^−1^. The produced glass samples may be employed as amorphous semiconductors in electronics and memory switching devices.

## Data Availability

The datasets used and/or analyzed during the current study available from the corresponding author on reasonable request.
